# Arthroscopic Fixation of Posterior Cruciate Ligament Tibial Avulsion Fractures With Single‐Suture and Dual EndoButtons via the Lateral Aspect of the Anterior Cruciate Ligament

**DOI:** 10.1002/atn2.70219

**Published:** 2026-09-05

**Authors:** Siqi Yang, Shengkun Wu, Zhikuan Li, Conglei Dong, Jiangtao Dong

**Affiliations:** ^1^ Hebei Medical University Third Hospital Shijiazhuang China

## Abstract

Arthroscopic techniques have gradually replaced open surgery as a routine method for treating posterior cruciate ligament tibial avulsion fractures. However, traditional arthroscopic surgery is associated with problems such as limited visualization, unstable fixation, and unsatisfactory reduction. By comparing various methods reported in the literature and integrating clinical experience, our team has developed an optimized technique to address current surgical challenges. Using dual posteromedial portals, we adopt a “single‐suture” method, in which the same epidural needle and a single suture are employed to encircle the posterior cruciate ligament via the lateral aspect of the anterior cruciate ligament, with dual EndoButtons applied for fixation. This technique not only precisely avoids the meniscofemoral ligaments and anterior cruciate ligament but also eliminates the need for separating the anterior cruciate ligament and posterior cruciate ligament, thus simplifying the procedure and reducing surgical injuries in the whole fixation process. It enhances fixation stability and surgical efficiency and preserves the posterior septum to promote postoperative functional recovery.

## Graphical abstract

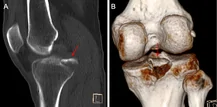

**VIDEO 1:** The anteromedial and anterolateral portals are established first. The arthroscope is inserted through the anterolateral portal to examine the meniscus. The lateral meniscus is stable, while a torn medial meniscus is repaired. A low posteromedial portal and a high posteromedial portal are then established. Under arthroscopic visualization, an epidural needle preloaded with a polydioxanone suture is inserted through the anterolateral portal into the interval between the PCL and the lateral wall of the intercondylar notch. The needle passes between the meniscofemoral ligament and the PCL, and 1 end of the polydioxanone suture is retrieved through the high posteromedial portal. The needle is then redirected beneath the ACL through the ACL‐PCL interval, and the other end of the polydioxanone suture is pulled out through the high posteromedial portal, forming a loop around the PCL. The PDS loop is used to shuttle 1 end of a FiberTape suture into the joint, and this procedure is repeated to introduce a second FiberTape. Debride the tissue between the posterior joint capsule and the tibia to expose the fracture fragment. After reduction of the avulsed fracture fragment, both ends of the same FiberTape are passed through the EndoButton for suspension fixation. Using a PCL reconstruction guide, 2 bone tunnels are drilled from the anterior tibial surface. The polydioxanone suture is passed through the bone tunnel and replaced with FiberTape. Both ends of the same FiberTape are passed through the EndoButton and tied securely to complete fixation. A postoperative physical examination is performed to ensure stable fixation of the right knee. (ACL, anterior cruciate ligament; PCL, posterior cruciate ligament.) Video content can be viewed at https://doi.org/10.1002/atn2.70219.

Posterior cruciate ligament avulsion fracture (PCLAF) is a rare intra‐articular injury of the knee joint, often caused by traffic accidents and sports trauma. Such injuries can lead to knee instability, limited mobility, and long‐term complications such as secondary osteoarthritis.^1^ For Meyers‐McKeever type III PCLAF (Figure 1),^2^ the fracture fragment is completely displaced and detached from the bone bed, making conservative treatment difficult for achieving anatomical reduction and stable fixation. Therefore, surgical reduction is usually required to restore joint stability.

**FIGURE 1:**
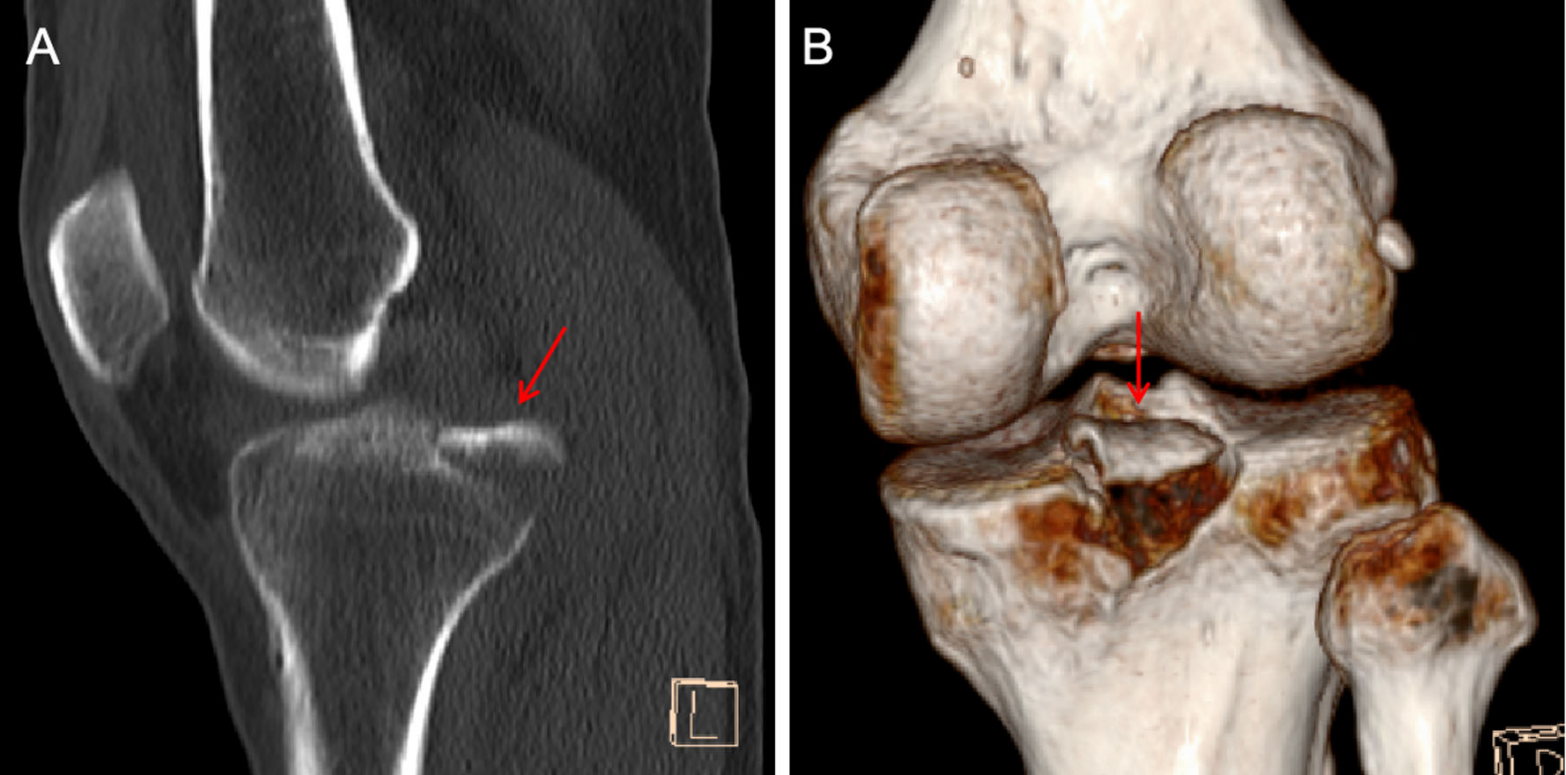
Preoperative (A) CT and (B) 3D reconstruction images of the right knee (red arrows indicate the avulsed bone fragment). (3D, 3‐dimensional; CT, computed tomography.)

With the continuous advancement of arthroscopic techniques, arthroscopic suture fixation has become the mainstream treatment for this injury due to its advantages of minimal invasion, clear visualization, and rapid postoperative recovery.^3^ The choice of arthroscopic technique depends on the fracture type, fragment size, displacement degree, fracture comminution, and associated injuries. Common clinical techniques include single‐row, double‐row, and suture bridge fixation,^4^ but traditional methods still face challenges regarding restricted visualization and operating space, difficulty in fracture fragment reduction and fixation, and complex suture passage and fixation techniques.^5^ To address these issues, we modified the traditional methods based on clinical practice and established the “single‐suture” technique with dual EndoButtons fixation via the lateral aspect of the anterior cruciate ligament (ACL) for the treatment of PCLAF.

This article will detail the surgical steps, technical pearls, and clinical value of this modified technique to provide a reference for clinical management.

## SURGICAL TECHNIQUE

The patient is placed in the supine position under general anesthesia. Knee joint physical examinations, including the anterior drawer test and Lachman test, are performed under anesthesia and compared with postoperative examination results to evaluate the surgical outcome.

**FIGURE 2:**
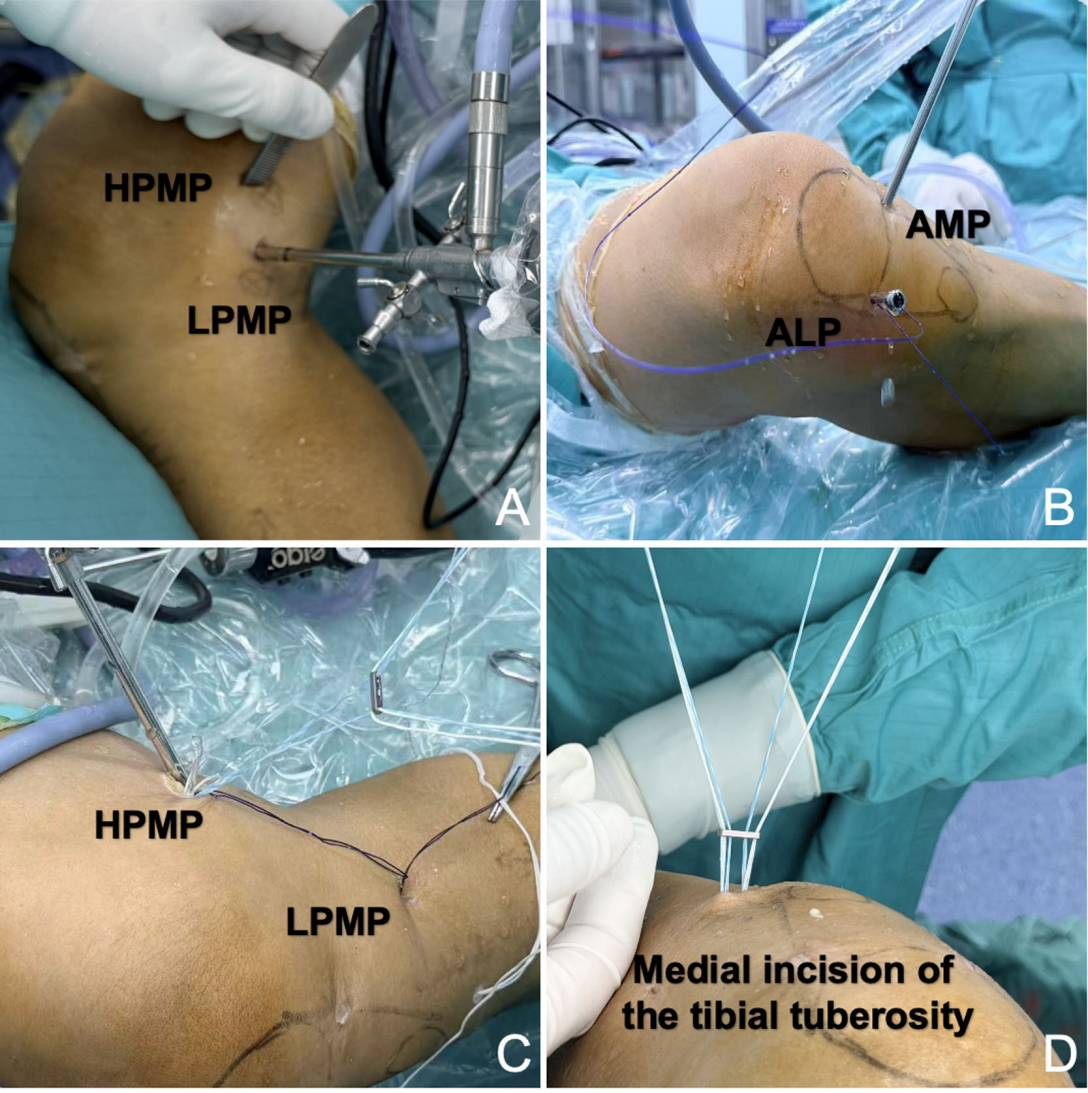
Extracorporeal schematic images illustrating portal establishment and EndoButton fixation in the right knee. (A) The HPMP is established through a longitudinal incision made 3 to 4 cm superior to the LPMP (figure‐of‐four position). (B) An epidural needle is inserted through the ALP (figure‐of‐four position). (C) The first EndoButton is anchored at the posterior tibia (supine position). (D) The second EndoButton is anchored at the anterior tibia (figure‐of‐four position). (ALP, anterolateral portal; AMP, anteromedial portal; HPMP, high posteromedial portal; LPMP, low posteromedial portal.)

**FIGURE 3:**
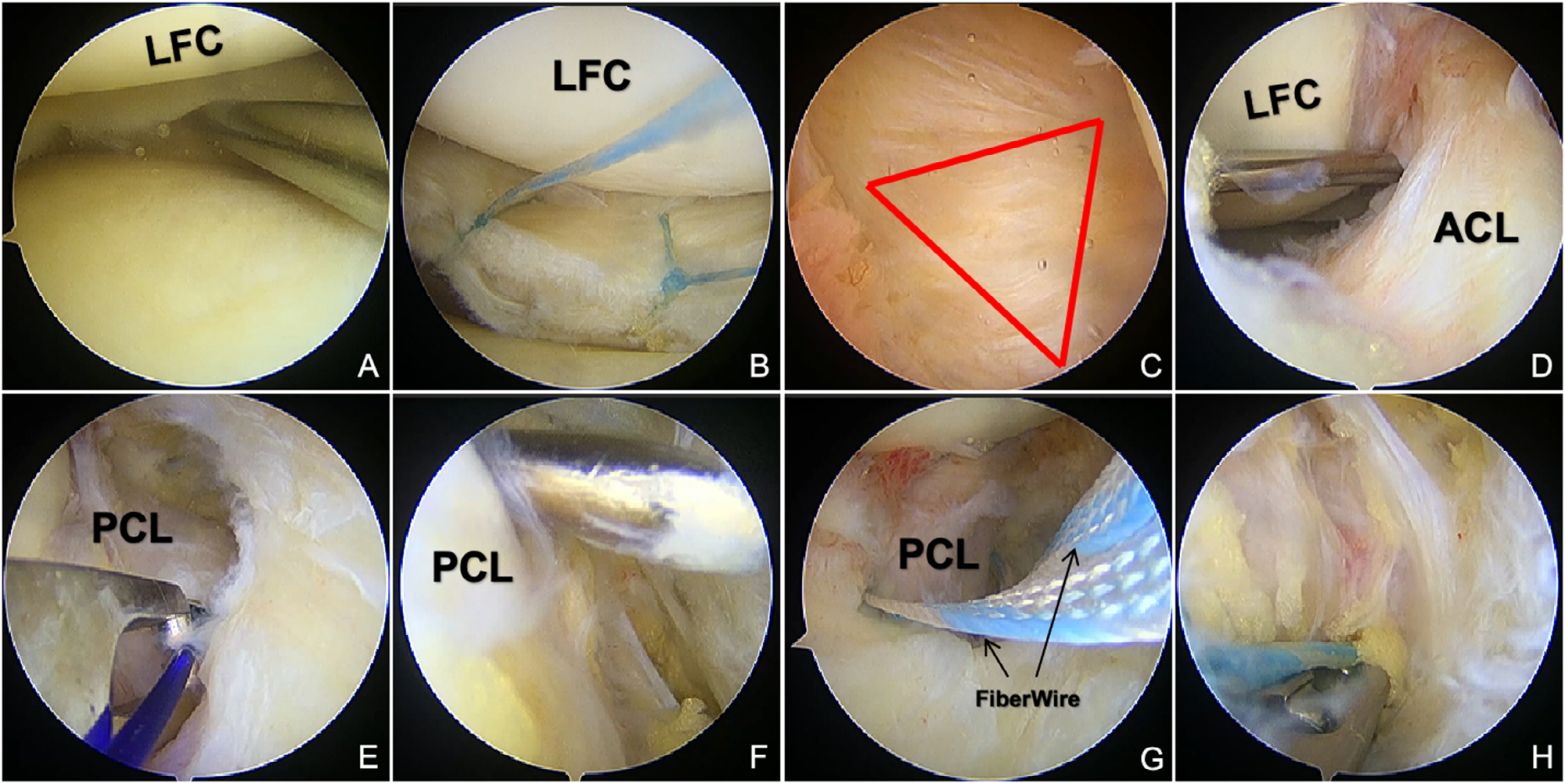
Arthroscopic views of the surgical procedure in the right knee. (A) The meniscus is probed, and the lateral meniscus is confirmed to be stable (figure‐of‐four position, anteromedial portal). (B) The torn medial meniscus is repaired using sutures (supine position, anteromedial portal). (C) Identification of the deformable “soft spot” in the triangular area bounded by the meniscal ramp zone, posterior femoral condyle, and medial head of the gastrocnemius fold (figure‐of‐four position, anteromedial portal). (D) An epidural needle is passed through the interval between the PCL and the lateral wall of the intercondylar notch (figure‐of‐four position, anteromedial portal). (E) One end of the polydioxanone suture is pulled out through the high posteromedial portal (figure‐of‐four position). (F) The epidural needle is passed through the ACL‐PCL interval beneath the ACL (figure‐of‐four position, anteromedial portal). (G) The PCL is encircled and fixed with fiber sutures (figure‐of‐four position, low posteromedial portal). (H) The avulsed bone fragment is reduced and fixed using an EndoButton (figure‐of‐four position, low posteromedial portal). (ACL, anterior cruciate ligament; LFC, lateral femoral condyle; PCL, posterior cruciate ligament.)

First, standard anteromedial and anterolateral arthroscopic portals are established. Routine arthroscopic inspection and joint cavity debridement are performed (Figure 2B), including removal of the hematoma, inspection of the meniscus (Figure 3A,B and Video 1), and exploration of the ACL and posterior cruciate ligament (PCL).

### Procedure

The arthroscope is advanced through the pathway between the PCL and the medial wall of the intercondylar notch into the posteromedial compartment. In the triangular area bounded by the ramp zone of the meniscus, posterior femoral condyle, and medial head of the gastrocnemius fold (Figure 3C), the most deformable “soft spot” is identified. A low posteromedial (LPM) portal is established using a scalpel. The arthroscope is withdrawn from the anterolateral portal and inserted into the posteromedial compartment through the LPM portal using a stitching stick. Under direct vision, a high posteromedial (HPM) portal is established 3 to 4 cm above the LPM portal (Figure 2A). With the LPM serving as the viewing portal and the HPM as the working portal, tissue between the PCL and the posterior capsule is debrided arthroscopically with a shaver, exposing the fracture fragment.

Under arthroscopic visualization, an epidural needle preloaded with a polydioxanone suture (PDS) is inserted into the space between the PCL and the lateral wall of the intercondylar notch through the anterolateral portal, advancing along the lateral aspect of the ACL (Figures 2B and 3D). After passing between the meniscofemoral ligament (MFL) and the PCL, 1 end of the PDS is pulled out through the HPM portal (Figure 3E). The epidural needle is then retracted and advanced again along the interval between the PCL and the lateral wall of the intercondylar notch, passing inferior to the ACL through the ACL‐PCL interval (Figure 3F). The other end of the PDS is also pulled out through the HPM portal, encircling the PCL (Figure 3G).

The loop of the PDS is used to shuttle 1 end of a FiberTape suture (Arthrex, Naples, FL) into the joint. The PDS loop is tightened to secure 1 end of the FiberTape, and the FiberTape is introduced into the joint by pulling the PDS. This procedure is repeated to shuttle the second FiberTape into the joint. After satisfactory reduction of the avulsed fragment, both ends of the same FiberTape are passed through the loops of an EndoButton (Smith & Nephew, London) (Figures 2C and 3H). The suture tension is adjusted to ensure the EndoButton closely adheres to the surface of the bone fragment at the predetermined fixation area, maintaining anatomical reduction.

Through the anteromedial portal, a PCL reconstruction locator (Smith & Nephew, London) is positioned at the inferolateral aspect of the tibial fracture line, closely adjacent to the distal reflection of the posterior joint capsule. A longitudinal incision approximately 2 cm in length is made on the medial side of the tibial tubercle, and a 4.5‐mm drill bit is used to create the first bone tunnel. After tunnel creation, an epidural needle is used to introduce the PDS into the joint, and the PDS loop is retrieved through the HPM portal using a suture retriever. The PDS loop is then used to pull both ends of the same FiberTape through the first bone tunnel. A second bone tunnel is established at the inferomedial aspect of the tibial fracture site, and the same steps are repeated.

**FIGURE 4:**
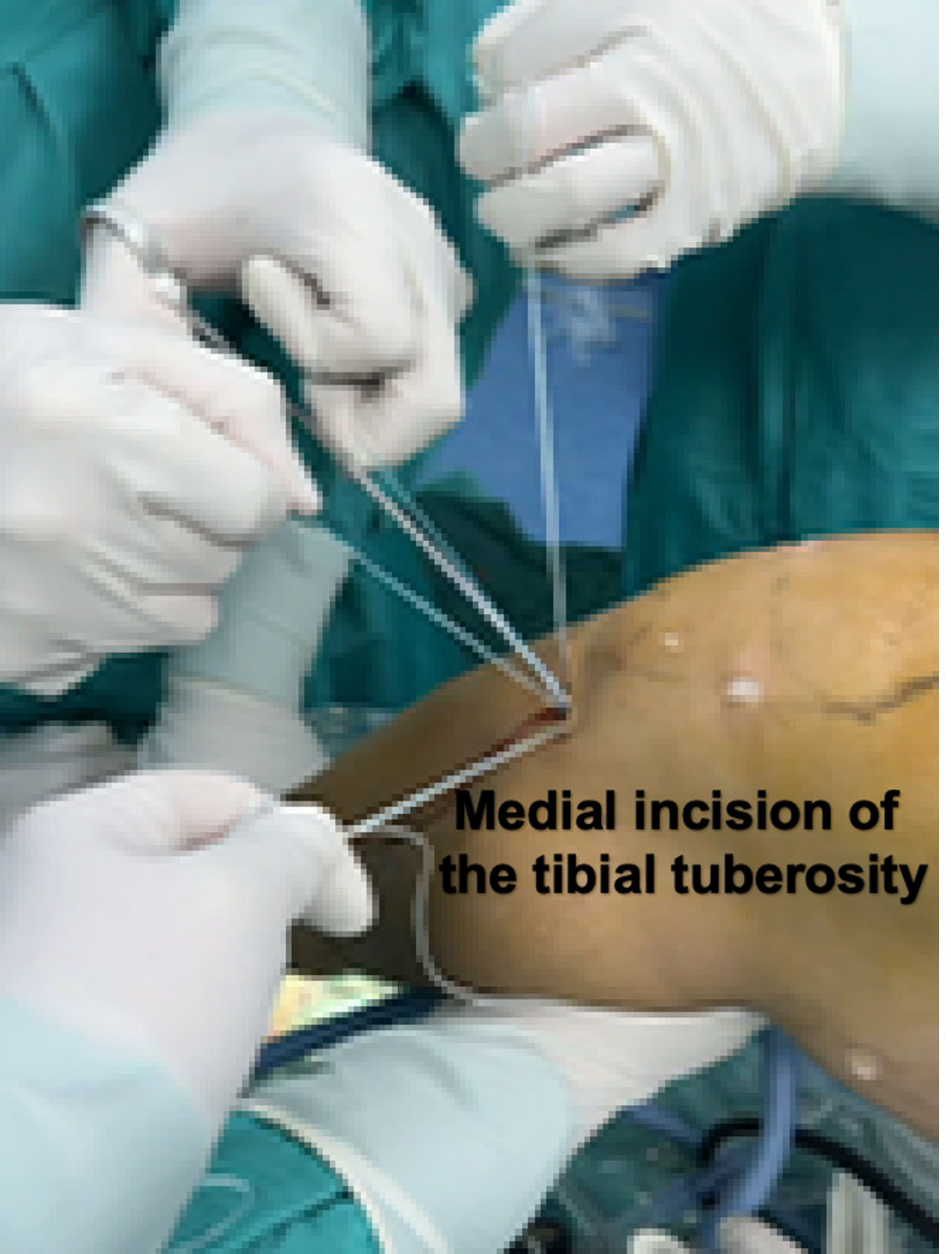
The fixation is performed while applying the “anterior drawer test” to the right knee (supine position).

**FIGURE 5:**
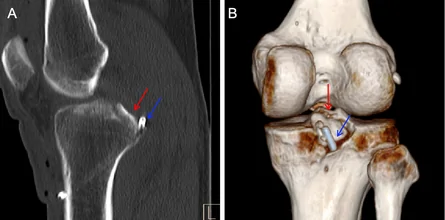
Postoperative (A) CT and (B) 3D reconstruction images of the right knee (red arrows indicate the avulsed bone fragment and blue arrows indicate the EndoButton used for fixation). (3D, 3‐dimensional; CT, computed tomography.)

After the 2 FiberTape sutures are retrieved separately through different bone tunnels, both ends of the same FiberTape are threaded through the 2 ends of the EndoButton for suspension fixation and secured using a knot pusher (Figure 2D). Finally, with the knee positioned at 90° flexion in neutral position, an anterior drawer force is applied to the tibia to restore physiological tension of the PCL (Figures 4 and 5), and the sutures are tied.

## DISCUSSION

This surgery adopts the double posteromedial portal composed of LPM and HPM. This approach preserves the posterior septum, maintains a large operating space and clear view of the posteromedial compartment, of the knee joint, and enables precise identification of anatomical structures such as the MFL, ACL, PCL, and fracture fragment.^6^ Furthermore, preserving the posterior septum structure can promote postoperative function recovery of PCLAF patients.^7^

The MFL includes the anterior and posterior MFL, originating from the posterior horn of the lateral meniscus, and inserting into the medial or lateral femoral condyle, lying adjacent to the PCL.^8^ In traditional PCLAF repair, there are 2 major challenges. First, separating the ACL from the PCL is a necessary and technically challenging step. The use of trocars in this tight space carries a high risk of iatrogenic injury to the substance of both ligaments. Second, when encircling the PCL, poor visualization or inaccurate operation can lead to the accidental encircle of the MFL. This not only compromises PCL fixation but may also cause abnormal movement of the lateral meniscus, leading to knee instability.^9^

**FIGURE 6:**
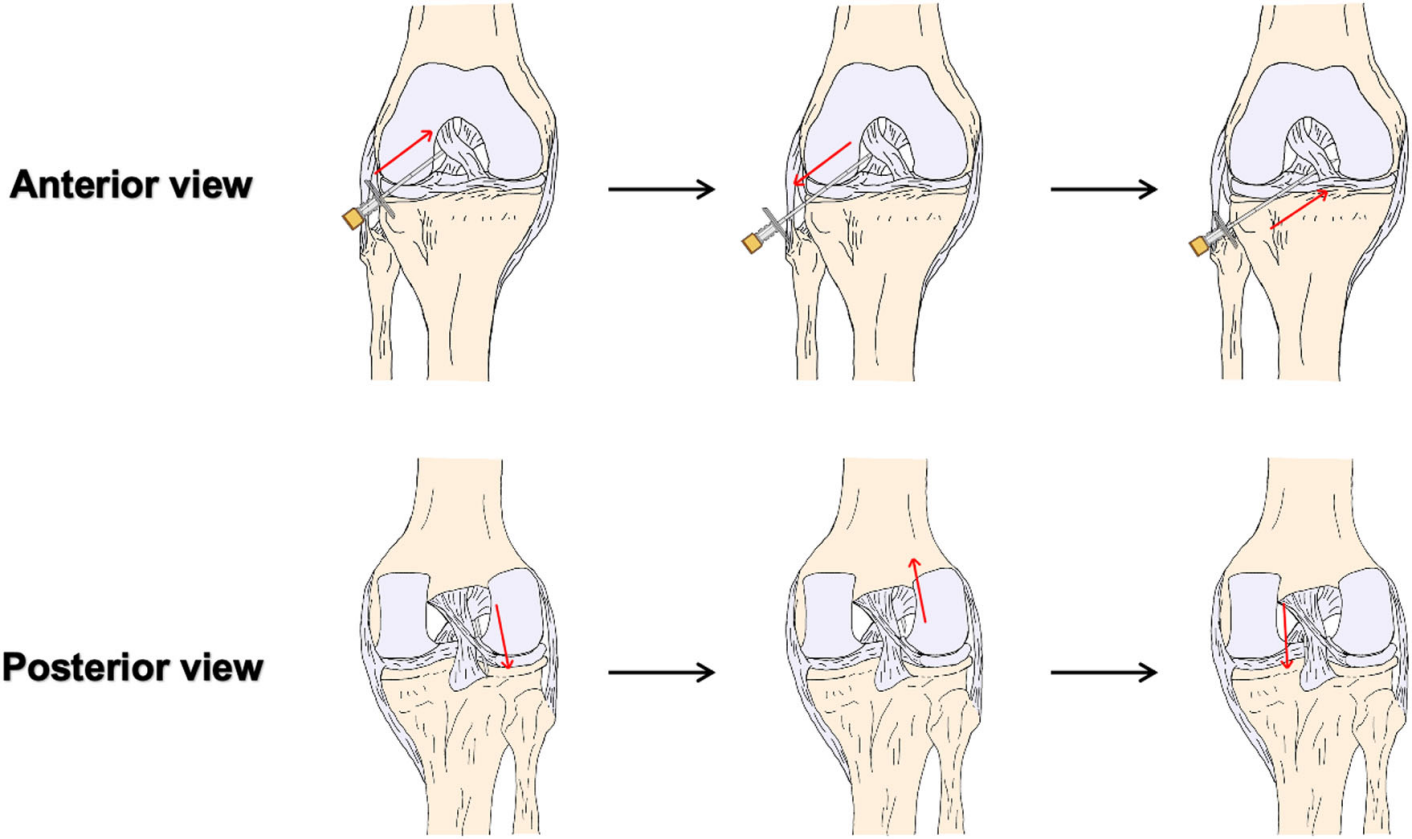
Schematic diagram illustrating the single‐suture technique in the right knee.

This “single‐suture” technique optimizes the operative pathway by using the same epidural needle sequentially through the “PCL‐lateral wall of intercondylar notch” space and the “ACL‐PCL” space beneath the ACL, eliminating the need for additional separation of the ACL and PCL, avoiding accidental injury to the ligaments, and simplifying the operation process of this surgery (Figure 6). Precise passage of the epidural needle through the “PCL‐MFL” interval ensures isolation of the PCL, effectively avoiding unintended entrapment of the MFL, and ensuring smooth suture shuttling. Additionally, passing through the “ACL‐PCL” interval can further prevent accidental looping of the ACL, reducing the risk of ACL injury.

Arthroscopic suture fixation for PCLAF provides good stability and functional recovery.^10^ Compared with traditional multisuture fixation methods, this “single‐suture” technique employs the same epidural needle that remains within the joint to complete the entire fixation process with only one suture. This avoids repeated looping errors, simplifies the procedure, reduces material and time consumption, and improves surgical efficiency. Moreover, the combined use of 2 EndoButtons provides a buttress effect exerting a compressive and restrictive load that ensures firm contact between the fragment and the fracture bed. This disperses stress, reduces the risk of reavulsion, and achieves more accurate anatomical reduction.^11^

During the procedure, when an epidural needle is advanced through the interval pathway, the surgeon should carefully and accurately identify the natural intervals among the ACL, PCL, and the MFLs to avoid inadvertent ligament penetration and iatrogenic injury. The needle trajectory should be maintained in a stable and controlled manner to minimize soft‐tissue trauma during manipulation. In addition, for cases involving severely comminuted avulsion fractures, extremely small fracture fragments, or poor bone quality, alternative or augmented fixation strategies should be considered according to the specific fracture characteristics. Finally, as this study is primarily a technical description, the applicability of this technique across different types of PCLAF and its long‐term clinical outcomes require further validation through large‐sample studies with extended follow‐up.

Table 1 summarizes the advantages and disadvantages of this technique, and Table 2 outlines technical pearls and pitfalls.

**TABLE 1:** Advantages and Disadvantages

Advantages
Dual posteromedial portals are utilized to maintain a clear view and adequate working space in the posteromedial compartment, while preserving the integrity of the posterior septum to promote postoperative recovery
Separation of the ACL and PCL is not required. The epidural needle is advanced through the interligamentous interval, thereby avoiding trocar manipulation that may cause ligament injury and preventing accidental looping of the meniscofemoral ligament and ACL, ensuring smooth suture passage and procedural safety
The “single‐suture” technique simplifies the surgical procedure, reducing material use and operative time, whereas the combined application of EndoButtons enables precise anatomical reduction of the fracture fragment, disperses stress, and minimizes the risk of reavulsion

Disadvantages
The dual posteromedial portal has a long learning curve, and there is a potential risk of injury to the saphenous nerve and great saphenous vein if standard portal placement techniques are not strictly followed
This technique requires a high level of anatomical recognition and precise control of epidural needle puncture, with a potential risk of inadvertent penetration of the ACL during the procedure
Compared with open reduction and internal fixation, there remains a potential risk of fragment displacement in cases with severely comminuted fragments or poor bone quality, and appropriate patient selection and fixation augmentation should be considered when necessary

ACL, anterior cruciate ligament; PCL, posterior cruciate ligament.

**TABLE 2:** Pearls and Pitfalls

Pearls
The dual posteromedial portal approach can reduce the risk of intraoperative injury to the posterior septum
Using an epidural needle allows nontrocar manipulation, which reduces the risk of iatrogenic ligament injury and prevents inadvertent looping of the ACL and the MFL

Pitfalls
The EndoButton may flip during traction when tension is applied for fragment fixation
The bone tunnel should be positioned inferior to the tibial avulsion fragment to ensure optimal fixation

ACL, anterior cruciate ligament; MFL, meniscofemoral ligament.

In conclusion, arthroscopic fixation of PCLAF using the “single‐suture” technique with dual EndoButtons fixation via the lateral aspect of the ACL represents a reliable and efficient surgical treatment option.

## DISCLOSURES

The author (J.D.) declares the following financial interests/personal relationships which may be considered as potential competing interests: J.D. reports funding from the National Natural Science Foundation of China and the Hebei Provincial Department of Science and Technology for this work. The other authors (S.Y., S.W., Z.L., C.D.) declare that they have no known competing financial interests or personal relationships that could have appeared to influence the work reported in this paper.

## FUNDING

This study was supported by National Natural Science Foundation of China (82471596), Natural Science Foundation of Hebei Province (H2025206050), Hebei Medical University (2022LCTD‐B25), Third Hospital of Hebei Medical University (CN) (ydsyqjjh202508) and Hengrui Hebei Innovative Development Medical Cooperation Program Project (CN) (HR202502051).

## References

[1] Gopinatth V , Mameri ES , Casanova FJ , et al. Systematic review and meta‐analysis of clinical outcomes after management of posterior cruciate ligament tibial avulsion fractures. Orthop J Sports Med. 2023;11:23259671231188383. 37724253 10.1177/23259671231188383 PMC10505349

[2] Li X , Ma Q , Zheng Q , et al. Modified arthroscopic repair of a posterior cruciate ligament tibial avulsion fracture improves IKDC and lysholm score compared to open reduction. J Orthop Surg Res. 2024;19:362. 38890683 10.1186/s13018-024-04851-4 PMC11184816

[3] Sundararajan SR , Joseph JB , Ramakanth R , Jha AK , Rajasekaran S . Arthroscopic reduction and internal fixation (ARIF) versus open reduction internal fixation (ORIF) to elucidate the difference for tibial side PCL avulsion fixation: A randomized controlled trial (RCT). Knee Surg Sports Traumatol Arthrosc. 2021;29:1251‐1257. 32712683 10.1007/s00167-020-06144-9

[4] Wang X , Zhang Z , Zi S , Wei W , Cheng B , Cao L . Biomechanical evaluation of three different fixation methods for treating displaced tibial avulsion fracture of the posterior cruciate ligament: A finite element analysis. Injury. 2025;56:112568. 40609241 10.1016/j.injury.2025.112568

[5] Chen G , Xu W , Chen Y , Wang W , Yu C . Classification and bundle‐weaving fixation for posterior cruciate ligament tibial avulsion fractures: Innovations and applications of key techniques. Orthop Surg. 2025;17:2943‐2959. 40878751 10.1111/os.70135 PMC12497554

[6] Vishwakarma NS , Gali JC , Gali JCF , LaPrade RF . Dual postero‐medial portal technique for posterior cruciate ligament tibial avulsion fracture fixations. Arthrosc Tech. 2021;10:e2229‐e2235. 34754728 10.1016/j.eats.2021.05.030 PMC8556587

[7] Shang Z , Jin L , Chen Z , et al. X‐shaped knot fixation and double posteromedial portals for the treatment of posterior cruciate ligament tibial avulsion fractures with retention of the posterior septum. Arthrosc Tech. 2024;13:102814. 38312887 10.1016/j.eats.2023.08.019 PMC10837810

[8] Corbi‐Aguirre F , Forriol F . Relationship of the cruciate and meniscofemoral ligaments with the knee osteology. An anatomical study. Rev Bras Ortop. 2023;58:85‐91. 10.1055/s-0042-1750073 PMC10038723 36969781

[9] Dzidzishvili L , Bi AS , Ostojic M , Chahla J . Combined lateral meniscus posterior root and meniscofemoral ligament injuries increase tibiofemoral forces and compromise rotational stability in ACL‐deficient and reconstructed knees: A systematic review and meta‐analysis of biomechanical studies. J Exp Orthop. 2025;12:e70227. 40182611 10.1002/jeo2.70227 PMC11966645

[10] Zhang L , Guo D . Clinical outcomes of arthroscopic suture fixation combined with loop plate vs. posterior approach open reduction and cannulated screw fixation for treating tibial avulsion fractures of the posterior cruciate ligament: A retrospective study. PeerJ. 2024;12:e18532. 39559331 10.7717/peerj.18532 PMC11572384

[11] Li Y , Liu JC , Wu J , et al. Biomechanical study of posterior cruciate ligament tibial arrest avulsion fracture fixation with triple tibial channel net sutures. Sci Rep. 2023;13:22980. 38151505 10.1038/s41598-023-50479-5 PMC10752874

